# Simultaneous Ultrasound and Heat Enhance Functional Properties of Glycosylated Lactoferrin

**DOI:** 10.3390/molecules25235774

**Published:** 2020-12-07

**Authors:** Zhipeng Li, Dexue Ma, Yiyang He, Siqi Guo, Fuguo Liu, Xuebo Liu

**Affiliations:** College of Food Science and Engineering, Northwest A&F University, Yangling 712100, China; zhipengli97@163.com (Z.L.); 15621552207@163.com (D.M.); yiyanghe2020@163.com (Y.H.); gsq22416@163.com (S.G.)

**Keywords:** ultrasound-assisted treatment, Maillard reaction, structural and functional properties

## Abstract

Protein-polysaccharide covalent complexes exhibit better physicochemical and functional properties than single protein or polysaccharide. To promote the formation of the covalent complex from lactoferrin (LF) and beet pectin (BP), we enhanced the Maillard reaction between LF and BP by using an ultrasound-assisted treatment and studied the structure and functional properties of the resulting product. The reaction conditions were optimized by an orthogonal experimental design, and the highest grafting degree of 55.36% was obtained by ultrasonic treatment at 300 W for 20 min and at LF concentration of 20 g/L and BP concentration of 9 g/L. The formation of LF-BP conjugates was confirmed by sodium dodecyl sulfate polyacrylamide gel electrophoresis (SDS-PAGE) and Fourier transform infrared (FTIR) spectroscopy. Ultrasound-assisted treatment can increase the surface hydrophobicity, browning index, 1,1-diphenyl-2-picryl-hydrazyl (DPPH) and 2,2’-azinobis-(3-ethylbenzthiazoline-6-sulphonate) (ABTS) free radicals scavenging activity of LF due to the changes in the spatial configuration and formation of Maillard reaction products. The thermal stability, antioxidant activity and emulsifying property of LF were significantly improved after combining with BP. These findings reveal the potential application of modified proteins by ultrasonic and heat treatment.

## 1. Introduction

Lactoferrin (LF), a member of the transferrin family, is an 80 kDa iron-binding glycoprotein added into nutritional supplements, infant formula, cosmetics and toothpaste [1]. LF has many advantages such as antimicrobial, antibacterial, antitumor and anti-inflammation activities and is often used as an emulsifier or essential component and as an amphiphilic milk protein in various delivery systems [2]. However, because of the instability (e.g., denaturation, aggregation) to the changes of pH and temperature, the application of LF as an emulsifier is limited [3]. Hence, chemical, physical and enzymatic modifications are widely used to improve the functional properties of food proteins. Unfortunately, the majority of chemical modifications of food proteins can cause health problem in some way, while enzymatic modifications are more expensive. Maillard reaction, a food-derived reaction that mostly happens between proteins and polysaccharides, performs easily without any extra chemical reagent and is widely used in protein modification [4]. The conjugation of proteins with polysaccharide is an efficient way to improve their functions, such as emulsibility, gel, and foaming properties [5]. Dry heating and wet heating are mainstreams of protein-polysaccharide grafting reactions, but both have shortcomings. Dry heating always takes a long time to form conjugates and the reaction extent is usually hard to control, which may result in excessive browning development [6]. Moreover, wet heating takes less time, but the higher reaction temperature can lead to protein aggregation [6]. Thus, it is essential to find an effective way to form protein-polysaccharide conjugates under more controlled conditions. 

Ultrasound treatment can be divided according to the frequency range of the power used into low-energy ultrasound (low power, low intensity), with frequency above 100 kHz at intensity below 1 W·cm^2^, and high-energy ultrasound, with intensity above 1 W·cm^2^ at frequencies between 20 and 500 kHz [7]. These two types have different applications in food modification. Low- and high-power ultrasonic treatments are usually utilized to improve food quality and food functional properties respectively [8]. Ultrasound-assisted Maillard reaction is increasingly applied in the preparation of glycosylated proteins [9,10,11]. Li et al. [12] prepared a covalent complex of peanut protein isolate and glucomannan by using sonication method and damp heating. Results showed that the grafting degree, solubility and emulsifying properties of the conjugate were significantly improved after ultrasonic treatment. Many kinds of carbohydrates (e.g., dextran, lactose, glucose, pectin, galactose, corn fiber gum) are commonly utilized in forming Maillard conjugates [5]. Beet pectin (BP), an effective stabilizer of oil-in-water (O/W) emulsions, is significantly different from the widely-used pectin [13]. Structurally, the existence of protein residues and phenolic esters is the main reason for the better properties of BP compared with other polysaccharides [14].

To the best of our knowledge, the effects of ultrasound and heat treatment on LF and glycated LF have not been investigated yet. In this study, LF and BP conjugates were prepared by high-intensity ultrasound treatment. The structural and functional properties of the conjugates were further studied compared with native LF, ultrasound-treated LF, mixture of untreated LF-BP and dry-heated LF-BP conjugates. 

## 2. Results and Discussion

### 2.1. Effect of Ultrasound Treatments on the Graft Reaction between LF and BP

Degree of graft (DG%) is often used to evaluate the extent of Maillard reaction. Glycation of LF is based on the reaction between the free amino group of the protein and the carbonyl group at the end of polysaccharide molecules [9]. Thus, the level of glycation grafting reaction can reflect the extent of Maillard reaction of the protein. 

#### 2.1.1. Effects of LF-BP Mass Ratio, Ultrasound Power and Ultrasound Time

**Figure 1:**
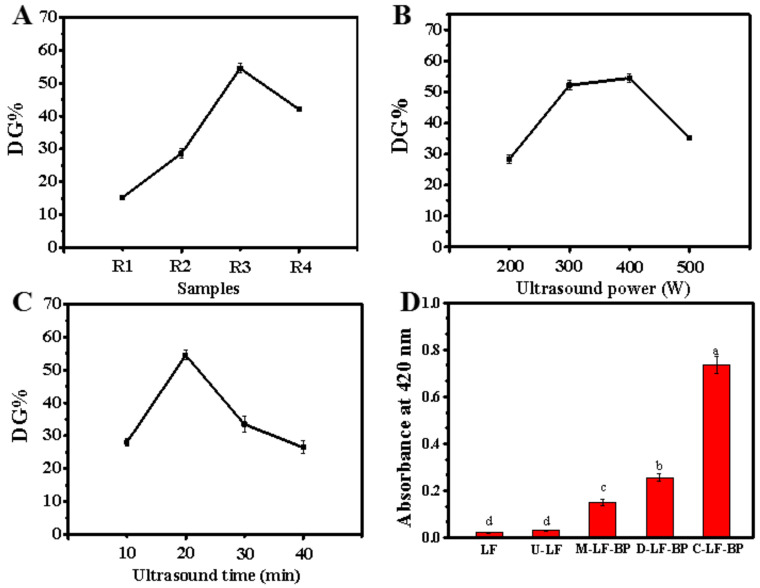
(**A**–**C**) Effects of different reaction conditions on the degree of graft of LF-BP conjugates. (**A**) LF to BP weight ratio (400 W; 20 min), R1: 2:0.3; R2: 2:0.6; R3: 2:0.9; R4: 2:1.2; (**B**) ultrasound power (LF:BP weight ratio 2:0.9 (R3), 20 min); (**C**) ultrasound time (LF:BP weight ratio 2:0.9 (R3), 500 W); (**D**) browning activity of LF, U-LF, M-LF-BP, D-LF-BP and C-LF-BP. LF: native lactoferrin; U-LF: ultrasound treated LF; M-LF-BP: mixture of untreated LF and BP; D-LF-BP: dry-heated LF and BP; C-LF-BP: ultrasound-assisted treated LF and BP. In Figure 1D, Bars with different letters are significantly different from each other (*p < 0.05*).

DG increases quickly first when the LF to BP mass ratio changes from 2:0.3 to 2:0.9 (Figure 1A). This suggests that with the increment of BP proportion, more reducing-end carbonyl groups are introduced to the system, which raises the possibility of the interaction. After meeting the saturation point, DG decreases, which is probably because of the decreased accessibility of the amino groups and the stereo-hindrance effect of the pectin. Due to the high pectin concentration, the sample with mass ratio of 2:1.2 is too viscous. In order not to cause damage to the ultrasonic instrument, mass ratio of 2:0.3, 2:0.6, 2:0.9 are used for the subsequent orthogonal test.

Ultrasound power is also a main factor of DG. DG increases rapidly with the growth of ultrasound power (Figure 1B). Because of its functions (e.g., mechanical mass transfer), heating and cavitation, ultrasound treatment can make the protein provide more free amino groups, which is beneficial to the Maillard reaction between protein and polysaccharide [15]. Moreover, local relative translational motions caused by ultrasound treatment can bring reactive groups closer [16]. After peaking at 400 W, DG decreased at 500 W. This is mainly because high intensity ultrasound induced formation of new protein aggregates which prevent the Maillard reaction [9].

The DG increases first, peaks at 20 min and then decreases quickly (Figure 1C). The evolution of ultrasound time indicates the extent of graft reaction, which may be the main reason for the increment in DG. However, the DG of samples decreases when reaction time exceeds 20 min, which is mainly because of the cross-linking between protein molecules [17]. Therefore, based on the above results, the extraction time of 10, 20, 30 min, power of ultrasound of 300, 400, 500 W, and LF to BP mass ratio of 2:0.3, 2:0.6, 2:0.9 were selected as the levels of the orthogonal test. 

#### 2.1.2. Orthogonal Analysis

Traditional comprehensive design involves 27 different experiments, while orthogonal table (L_9_) testing only needs nine experiments. The results of DG% are shown in Table 1. According to the range analysis, the effects of the three factors on the DG of LF-BP conjugates rank as: LF to BP mass ratio (A) > ultrasonic time (B) > ultrasonic power (C). Within the designed ranges, the optimal condition is A_3_B_2_C_1_, which means the LF to BP mass ratio of 2:0.9, ultrasonic treatment time of 20 min, and ultrasonic power of 300 W.

#### 2.1.3. Confirmation Experiment

To check whether the predicted optimal condition is consistent with the actual results, confirmation experiment was conducted at the LF to BP mass ratio of 2:0.9, ultrasonic treatment time of 20 min and ultrasonic power of 300 W. Results showed that DG between LF and BP was 55.36%, which was higher than that at other levels. Therefore, the investigation proves that the optimal levels arrived for the significant factors are correct.

### 2.2. Browning Index

Browning index is important in evaluating the Maillard reaction products (MRPs). The formation of complex from sugar and protein in Maillard reaction takes three major stages, which are early, intermediate and final stages. In the final stage, the melanoidin generated from Maillard reaction can cause browning in MRPs, which is considered as the sign of Maillard reaction and is intensified with the increasing degree of reaction. The absorbance at 420 nm used as the browning index is widely applied for identifying the amount of high-molecular-weight melanoidins [18]. The browning index data of LF, U-LF, M-LF-BP, D-LF-BP and C-LF-BP are shown in Figure 1D. Clearly, the absorbance of native LF before and after ultrasound-assisted treatment did not significantly differ, while the absorbance at 420 nm of M-LF-BP increased, which could be attributed to the introduction of BP. Compared with M-LF-BP, both D-LF-BP and C-LF-BP showed an increment in the absorbance at 420 nm, indicating the formation of the final MRP-melanoidin and proving that Maillard reaction occurred in both the dry-heating group and the ultrasound-assisted group. However, C-LF-BP had a higher absorbance at 420 nm than that of D-LF-BP, which may be because high ultrasonic power increases local temperature of the solution and then the degree of browning [9].

### 2.3. SDS-PAGE

**Figure 2:**
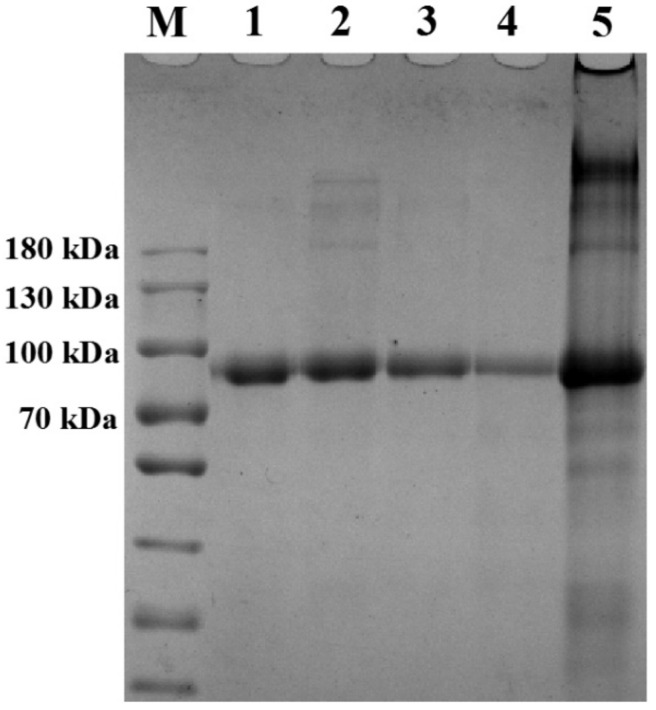
SDS-PAGE of LF, U-LF, M-LF-BP, D-LF-BP and C-LF-BP. M: Marker (10–180 kDa); lane 1: LF; lane 2: U-LF; lane 3: M-LF-BP; lane 4: D-LF-BP; lane 5: C-LF-BP.

SDS-PAGE is an efficient way to prove the formation of covalent link between proteins and polysaccharides during Maillard reaction, and is widely used in previous studies [9,19]. The SDS-PAGE patterns of LF, U-LF, M-LF-BP, D-LF-BP and C-LF-BP are shown in Figure 2. 

New bands appeared in lane 2 at above 100 kDa when LF was processed by ultrasound-assisted treatment, which indicates the formation of cross-links between protein molecules. This phenomenon agrees with the results in Section 2.1.1. The band of the untreated mixture (lane 3) is similar with that of native LF (lane 1). An obvious band appears in lane 5 at the top of the stacking gel, which indicates the formation of covalent bonds between the amino groups of LF and the carbonyl groups of BP, and mainly suggests the formation of conjugates after Maillard reaction. This phenomenon is consistent with previous studies [19,20]. Compared with C-LF-BP (lane 5), D-LF-BP (lane 4) does not show any significant band at the top of the gel, indicating there is no obvious formation of conjugates, which proves that ultrasound-assistant treatment is more effective than heating treatment in forming MRPs.

### 2.4. Fluorescence Spectra

**Figure 3:**
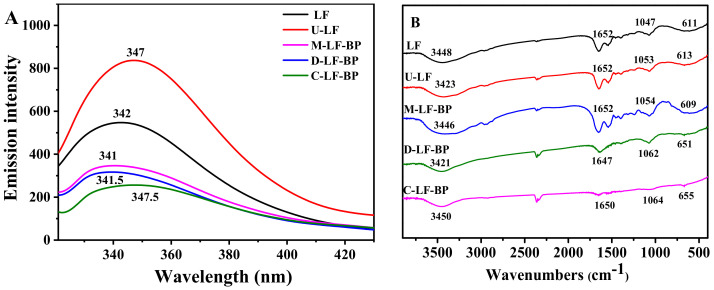
(**A**) Fluorescence intensity and (**B**) FTIR spectra of LF, U-LF, M-LF-BP, D-LF-BP and C-LF-BP.

Fluorescence emission of tryptophan is extremely sensitive to changes of the surrounding environment. Fluorescence spectroscopy mainly characterizes the changes in tryptophan surroundings of proteins, which could be a sensitive index of alteration in protein conformation and amino acid loss after Maillard reaction [21]. It can be described as a great indicator of the conformational transition of proteins [22]. As shown in Figure 3A, the fluorescence intensity of U-LF significantly increased, while that of C-LF-BP decreased. The absorption peaks of both U-LF and C-LF-BP red-shifted at the excitation wavelength of 295 nm, suggesting that U-LF and C-LF-BP tend to be more hydrophobic [23]. The fluorescence intensity increment in U-LF indicates that ultrasound treatment can modify the structure of LF and expose the tryptophan residues on the surface [10]. Compared with native LF, the fluorescence of M-LF-BP decreases significantly, indicating that the hydrophobic environment is more likely to surround the tryptophan residues of LF [24]. After ultrasound-assisted covalent binding with BP, the fluorescence intensity of LF decreased, without shift compared with U-LF, and was lower than that of D-LF-BP. This result suggests that ultrasound-assisted treatment can modify the side chains of the protein in tertiary structure, rather than entirely destroying its native structure, and shows a stronger effect in modifying LF. Similar result was discovered in the glycation of bovine serum albumin [25].

### 2.5. FTIR

As an effective means of macromolecular substance analysis, FTIR is widely used to analyze the compositions of macromolecular polymers and secondary structure proteins according to energy absorption and intramolecular atomic vibration. The characteristic bands of proteins in the infrared region consist of three parts: bands at 1600–1700 cm^−1^ defined as amide I band which correspond to C-O and N-H bending; bands at 1450–1550 cm^−1^ known as the amide II bands of protein structures [26]; bands at 1200–1450 cm^−1^ associated with the stretching vibrations of C-N and N-H bending which are known as amide III group [27]. 

The FTIR spectra of the LF, U-LF, M-LF-BP D-LF-BP and C-LF-BP are shown in Figure 3B. The characteristic bands of native LF at 3448 and 1652 cm^−1^ are mainly attributed to N-H and C-O. Compared to native LF, spectra of U-LF show a blue shift at 3423 cm^−1^, indicating the N-H or hydrogen bonding is broken and ultrasonic treatment can affect the structure of LF.

Moreover, FTIR spectra of C-LF-BP show a red shift at 3450 cm^−1^, suggesting the -OH or -NH_2_ of LF may be involved in the binding [28]. The absorption intensities of both D-LF-BP and C-LF-BP at 1600–1700 cm^−1^ and 1200–1450 cm^−1^ (Figure 3B), which are related to amide I and III groups, are lower than those of the M-LF-BP, which is mainly due to the loss of NH_2_ groups and carbonyl groups during the glycation. Moreover, the absorption intensities of C-LF-BP at the wavelength in amide I, II, and III groups are lower than that of D-LF-BP, indicating that ultrasound-assisted treatment can involve more NH_2_ and carbonyl groups in the reaction.

### 2.6. TGA

**Figure 4:**
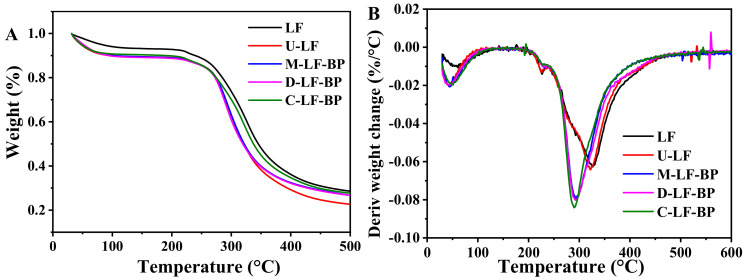
(**A**) Weight and (**B**) deriv weight change of LF, U-LF, M-LF-BP, D-LF-BP and C-LF-BP.

Thermogravimetric (TG) analysis is able to determine temperatures and rates of pyrolysis, while differential thermal gravimetric (DTG) curves show the exothermicity or endothermicity of the reactions that accompany the pyrolysis and combustion, which are valuable information in chemically assessing the thermal decomposition of MRPs [29]. The TG and DTG results of LF, U-LF, M-LF-BP, D-LF-BP and C-LF-BP are shown in Figure 4A. As shown by TG curves, the decomposition process can be divided into two stages in 30–600 °C. The first stage at 30–150 °C is mainly due to the loss of adsorbed and bound water. It is obvious on the DTA curve that the maximum moisture loss occurs at about 90 °C. However, the TG and DTG results of native LF show its least weight loss occurs within 30–150 °C. Because of the freeze-drying, the other four samples display a fluffy structure, which can enhance the water absorption on the surface of the materials. The second stage occurs at 150–350 °C, at which the sample experiences the maximum decomposition and weight loss rate, which indicates the degradation of polymers [30]. The degradation of the conjugates is mainly due to the decomposition of LF and BP. As for LF, the degradation is related to the non-covalent electrostatic bonds, and hydrophobic interaction decomposing, and then covalent bonds of amino acid residues are broken with the temperature rise [29].

At 500 °C, U-LF showed the lowest remaining polymer amount, which proved its inferior thermal stability to LF. Moreover, the residual amount of C-LF-BP was higher than those of dry-heating treated and physically-mixed LF and BP, while D-LF-BP did not show much increment compared to M-LF-BP. These results further prove the occurrence of Maillard reaction and the better effect of ultrasound-assisted treatment on Maillard reaction than that of dry-heating treatment. In addition, maximum weight loss temperatures (T_max_) of LF, U-LF, M-LF-BP, D-LF-BP and C-LF-BP are 326, 322, 295, 292, and 286 °C, respectively (Figure 4B). T_max_ of the MRPs is lower than those of only LF, and that of C-LF-BP is the lowest, which further prove the formation of easily thermally decomposed MRPs and the better effect of ultrasound-assisted treatment on the Maillard reaction. This result coincides with that of another study [31].

### 2.7. Surface Hydrophobicity (H_0_) of Proteins in Conjugates

**Figure 5:**
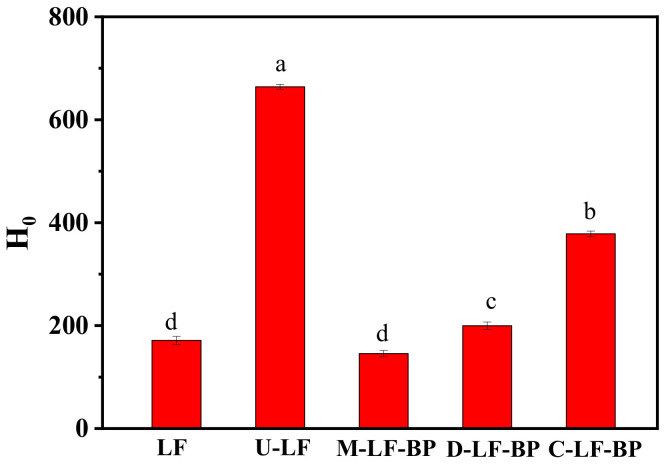
Surface hydrophobicity of LF, U-LF, M-LF-BP, D-LF-BP and C-LF-BP. Bars with different letters are significantly different from each other (*p < 0.05*).

H_0_ indicates the amount of hydrophobic groups on the surface of protein molecules in a polar water environment and critically affects protein functionality closely related to its functional properties, which closely relate to the emulsifying capacity and emulsion stability of MRPs [21]. The H_0_ of ultrasonic samples significantly increased compared to non-ultrasonic samples (*p* < 0.05) (Figure 5), indicating that ultrasonic treatment can significantly increase the H_0_ of LF. This result is consistent with the study by Chen et al. [32], which reveals that ultrasonic treatment can cause an increase in surface hydrophobicity of soybean isolate. The reason is that the hydrophobic regions in the peptide chains are exposed after cavitating caused by ultrasound treatment [33]. In addition, the H_0_ of both D-LF-BP and C-LF-BP significantly increased compared to native LF. Moreover, H_0_ of C-LF-BP was higher than that of D-LF-BP, which is possibly because ultrasound-assisted treatment compared with heating treatment can expose more hydrophobic clusters on the molecular surface, leading to the increase of H_0_. Compared with U-LF, the H_0_ of C-LF-BP was much lower even in the same ultrasound condition, indicating that more attachment of -OH in saccharide reduces the surface hydrophobicity [9].

### 2.8. Antioxidant Activities

**Figure 6:**
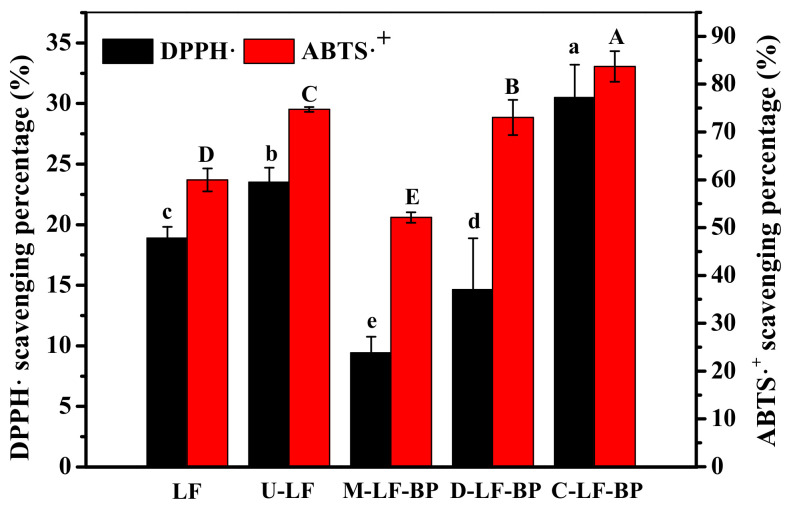
DPPH and ABTS free radicals scavenging abilities of LF, U-LF, M-LF-BP, D-LF-BP and C-LF-BP. Bars with different letters are significantly different from each other (*p < 0.05*).

The abilities of the samples to scavenge DPPH and ABTS free radicals were detected (Figure 6). 

Compared with native LF, the scavenging rate and reduction ability of LF after ultrasonic pretreatment significantly increased. The structure of the substrate protein moderately influences the antioxidant activity of proteolysates [34]. The fluorescence spectroscopy and FTIR spectra demonstrate that the cavitation and mechanical action of ultrasound-assisted treatment can disrupt the dense structure of the protein, expose its hydrophobic sites (e.g., tryptophan inside the protein molecule), and thereby increase its surface hydrophobicity. In comparison, tryptophan can provide hydrogen atoms to increase the antioxidant activity of the substance by terminating the free radical chain reaction [35]. 

According to Figure 6, the scavenging rate and reduction ability of both D-LF-BP and C-LF-BP are higher compared with M-LF-BP, and the activity of C-LF-BP is the highest among these groups. This result indicates that the Maillard reaction with BP can improve the DPPH and ABTS free radicals scavenging abilities of LF. Moreover, the scavenging ability is strengthened with the enhancement of reaction conditions, which is mainly because hydroxyl groups and/or reducing pyrrole and furan groups existing in MRPs can unleash antioxidant activity and provide hydrogen atoms [36]. Also, the higher ABTS free radical scavenging activity of MRPs can be attributed to the higher content of MRPs and some newly-formed peptides. Melanoidins (a brown polymer produced by heating polypeptides in the presence of reducing sugars) and free amino acids or peptides in MRPs can donate hydrogen atoms to ABTS free radicals [37]. Therefore, the samples exhibit high antioxidant activity.

### 2.9. Emulsion Activity and Stability

**Figure 7:**
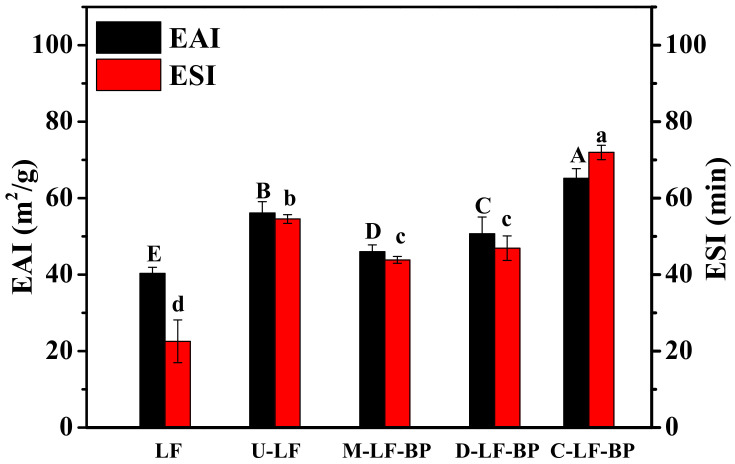
EAI and ESI of LF, U-LF, M-LF-BP, D-LF-BP and C-LF-BP. Bars with different letters are significantly different from each other (*p < 0.05*).

Emulsions are physiochemically unstable systems and can be separated into two immediate- or slow-immiscible phases. The combination between the functionality of protein (absorption to the oil-water interface) and the characteristic property of polysaccharides (solubility in aqueous phase medium) can significantly increase the stability of emulsions [27]. The emulsifying activity index (EAI) and emulsifying stability index (ESI) of LF, U-LF, M-LF-BP and C-LF-BP are shown in Figure 7. Clearly, the EAI and ESI of U-LF are significantly higher than those of LF, which is consistent with a previous study [38]. Because of its mechanical effects associated with cavitation, ultrasound treatment may break the structure of molecules, which increases the molecular mobility and much quickens the sample absorption at the oil/water interface. In addition, EAI and ESI of the untreated mixture increased compared with native LF, revealing a strong steric repulsion can be formed after the addition of BP, which is conducive to increasing the emulsifying activity of M-LF-BP. This result is consistent with the result by Zhong et al. [19] that the addition of *P. ostreatus* β-glucan can improve the emulsibility of original oat protein isolate. Furthermore, a combination of adsorption ability of protein moiety and the high hydrophilicity of polysaccharide leads to the formation of a strong solvated layer near the oil-water interface, which confers steric stabilization to emulsion oil droplets [16]. Moreover, both EAI and ESI of native LF were considerably improved after covalent binding with BP, which is mainly because protein molecules can expose more hydrophobic groups and gradually appear new balance values of hydrophobic and hydrophilic groups consents [10]. However, the EAI and ESI of D-LF-BP were lower than those of C-LF-BP even though MRPs were formed in all these groups. The reason for this phenomenon is that ultrasound-assisted treatment is more effective than heating treatment in modifying Maillard reaction, which is consistent with previous results.

## 3. Materials and Methods

### 3.1. Materials

LF powder was purchased from Westland Milk Products (Hokitika, New Zealand). According to the manufacturer’s specifications, this product contained 98.9% proteins, 0.6% moisture, and 0.5% ash. BP was purchased from Guangzhou Hongyuan Food Additive Co., Ltd. (Guangzhou, China), which contained 65% galacturonic acid and had molecular weight of 45 kDa, and 55% degree of esterification (DE). Corn oil was obtained from Shandong Luhua Group Co. Ltd. (Shandong, China). All other reagents were of analytical grade.

### 3.2. Preparation of LF-BP Conjugates

Single-factor tests conducted to optimize the preparation process of the LF-BP conjugates. The main factors were the LF to BP mass ratio, ultrasound time, and ultrasound power. Briefly, LF and BP were dissolved in deionized water and then gently stirred for 2 h. The solution was adjusted to pH 7.0 by using 1 M NaOH and HCl. The solution was treated by a JY92-IIN ultrasound processor model (Shanghai HuXi Industry Co. Ltd., Shanghai, China) with a sequence of 2 s of sonication and 2 s of rest at 90 °C. Subsequently, the solution was cooled to 25 °C and dialyzed at 4 °C for 2 days with interception molecular weight of 8 k–12 kDa. After that, the solution was lyophilized and stored at −20 °C. Effects of LF to BP mass ratio (2:0.3, 2:0.6, 2:0.9, 2:1.2), ultrasound time (10 to 40 min) and ultrasound power (200 to 500 W) on the Maillard reaction were investigated. The mass ratio was selected based on a previous study [39] with slight modification.

On the basis of the single-factor tests, L_9_ (3^3^) orthogonal test was designed, the best conjugating condition was selected via measuring the degree of graft (DG). Native LF (LF), ultrasound-treated LF (U-LF), mixture of untreated LF-BP (M-LF-BP) and dry-heated LF-BP (D-LF-BP) were also prepared as controls in the same way.

### 3.3. Measurement of DG

DG was determined based on the o-phthaldialdehyde (OPA) method [40]. OPA (80 mg) was dissolved into 2 mL of 95% methanol and mixed with 50 mL of a 100 mM sodium tetraborate buffer solution (pH 9.7), 5 mL of 20% (*w*/*w*) sodium dodecyl sulfate (SDS) and 200 μL of 2-mercaptoethanol. The resulting solution was mixed and diluted with distilled water to a final volume of 100 mL to form the OPA reagent. The sample dispersion (2 g/L, 200 μL) was incubated with 4 mL of the OPA reagent for 5 min at room temperature and then the absorbance at 340 nm was measured using a spectrophotometer to determine the free amino content. In the meantime, 200 μL of distilled water was mixed with 4 mL of the OPA reagent as a blank control. DG% was calculated as follows:

DG (%) = (A_0_ − A_1_)/A_0_ × 100%

where A_0_ and A_1_ are the absorbance values before and after glycation with BP respectively.

### 3.4. Browning Index 

The browning of samples was measured according to a given method [9] with slight modification. The samples were diluted in advance with 1 mg/mL SDS to the concentration of 2 mg/mL. The browning index was evaluated by measuring the absorbance at 420 nm on a UV-mini-1240 spectrophotometer (Shimadzu, Kyoto, Japan). 

### 3.5. Characterization of LF-BP Conjugates 

#### 3.5.1. SDS-PAGE

SDS-PAGE was performed using a reported method [27] with slight modifications. The test was carried out using 5% stacking gel and 12% separating gel. The samples were heated for 2 min in boiling water before electrophoresis. Commassie bright blue R-250 staining was chosen to explore the protein performance on the gel sheets. 

#### 3.5.2. FTIR Spectroscopy 

Information about the nature of the molecular interactions in the conjugates was obtained using a Vertex 70 FTIR instrument (Bruker, Ettlingen, Germany). The mixture of the sample and KBr at the ratio of 1:100 (1 mg:100 mg) was compressed to form the discs. All samples were scanned three times and measured at 4000–400 cm^−1^.

#### 3.5.3. Determination of Intrinsic Fluorescence Emission

Fluorescence was measured using a fluorescence spectrophotometer (LS55, PerkinElmer, MA, USA) according to a reported method [41] with slight modifications. Intrinsic fluorescence was measured at 0.2 mg/mL of the samples. The excitation and emission slit widths were both 10 nm while the scanning conditions were set at 295 nm (excitation) and 300–420 nm (emission).

#### 3.5.4. Thermal Gravimetric Analysis (TGA)

Thermal properties were analyzed by an STA7200RV TGA device (Hitachi Group, Japan) according to a reported method [42] with slight modifications. The thermal properties were studied under nitrogen atmosphere with a flow rate of 50 mL/min. At each time, a certain amount of a sample (3–5 mg) was heated from 30 to 600 °C at the rate of 10 °C/min. Weight loss of the sample was measured as a function of temperature.

#### 3.5.5. Surface Hydrophobicity (H_0_)

H_0_ was determined by using 1,8-aniline naphthalene sulfonate (ANS) as a fluorescent probe according to a previous study [9] with some modifications. ANS solution (20 μL, 8.0 mmol/L) was added to 4 mL of 0.05, 0.1, 0.2, 0.5, or 1 mg/mL protein solution prepared by phosphate buffer solution (PBS, 10 mmol/L, pH 7.0). Fluorescence intensity (FI) was measured using fluorescence spectrophotometry at 390 nm (excitation) and 470 nm (emission), both with slit width of 5 nm. The initial slope of the FI and protein concentration curves was used as the exponent of H_0_. 

### 3.6. Functional Properties of the Conjugates 

#### 3.6.1. DPPH Free Radical Scavenging Activity 

The antioxidant capacity of the samples was evaluated by measuring the DPPH radical scavenging capacity according to Yang et al. [43] with some modifications. A 0.1 M DPPH solution was prepared with 75% ethanol. The control group was prepared by mixing 1 mL of diluted water with 1 mL of the DPPH solution. Then 1 mL of different samples (1 mg/mL) were added into 2 mL of the fresh DPPH solution, kept at room temperature in the dark for 30 min and centrifuged at 1000× *g* for 10 min. Then the absorbance at 517 nm was measured using a UV-visible spectrophotometer. The DPPH radical scavenging activity was calculated as follows:

DPPH free radical scavenging capacity (%) = (A_control_ − A_sample_)/A_control_ × 100%

where A_sample_ and A_control_ are the absorbance values of the sample and control solutions, respectively.

#### 3.6.2. ABTS Free Radical Scavenging Activity 

The ABTS radical scavenging activity of samples was determined based on a previous method [44] with slight modification. An ABTS stock solution was prepared by mixing 7 mmol/L ABTS aqueous solution with a 2.45 mmol/L K_2_S_2_O_8_ solution and reacted in the dark for 12 h. Before the experiment, the ABTS stock solution was diluted with methanol until the absorbance at 734 nm reached 0.700 ± 0.02. Then 1 mL of the extract was diluted to a certain concentration, added with an ABTS solution at 1:3 (*v*/*v*), vibrated for 20 s, and reacted for 60 min. The absorbance at 734 nm was measured. The ABTS radical scavenging activity (%) was calculated as follows:

ABTS free radical scavenging capacity (%) = [1 − (A_2_ − A_1_)]/A_0_ × 100%

where A_0_, A_1_ and A_2_ are the absorbance values of the mixture of deionized water and ABTS solution, the mixture of deionized water and a sample, and the mixture of a sample and ABTS solution, respectively.

#### 3.6.3. Emulsifying Activity and Stability

The EAI and ESI were measured according a previous method [16] with some modifications. Briefly, a 0.2% (*w*/*v*) protein sample was dissolved in PBS (0.2 M, pH 7.5) and stirred at room temperature for two hours. The O/W emulsions were obtained by homogenizing 1 mL of corn oil and 3 mL of a 0.2% (*w*/*v*) protein sample solution at 20,000× *g* rpm for 1 min using an Ultra-Turrax device (T25, IKA Laborechnik, Staufen, Germany). Then 50 μL of emulsion was added into 5 mL of PBS (0.2 M pH 7.5) with 0.1% SDS (*w*/*v*). The absorbance of the emulsion was measured at 500 nm at 0 min (A_0_) and 10 min (A_10_). EAI and ESI were calculated as follows:

EAI (m^2^) = 2*T*A_0_ × dilution factor/*c* × Φ × *L* × 10000

ESI (min) = A_0_/(A_0_ − A_10_) × 10

where *T* = 2.303, dilution factor = 1000, *c* is the weight of protein per volume (g/mL), *L* is the width of the optical path (0.01 m), and Φ is the oil volumetric fraction (0.25).

### 3.7. Statistical Analyses

All experiments were performed in triplicate and the data were analyzed by one-way variance of analysis (ANOVA) using SPSS 17.0 (SPSS Inc., Chicago, IL, USA). Statistical significance analysis (*p* < 0.05) was determined using Duncan’s test.

## 4. Conclusions 

Ultrasound-assisted treatment significantly enhances the covalent binding between LF and BP. The best conditions for forming LF-BP conjugates were achieved using ultrasound treatment at 300 W for 20 min, in which the DG% between LF (2 wt%) and BP (0.9 wt%) was 55.36%. SDS-PAGE, FTIR, fluorescence spectroscopy, and results of browning index proved the formation of the LF-BP conjugates. Maillard reaction between LF and BP changed the structure of LF, which significantly enhanced its thermal stability. After covalent binding with BP, LF showed an increment in surface hydrophobicity and emulsibility. This study is helpful for developing a framework of utilizing ultrasound-assisted glycation to modify LF and for extending the applications of LF into the food industry.

## Figures and Tables

**Table 1:** Orthogonal experimental arrangement and test results.

Test Number	A: LF to BP	B: Ultrasonic	C: Ultrasonic	Degree of Graft (%)
	Mass Ratio	Time (Min)	Power (W)	
1	1 (2:0.3)	1 (10)	1 (300)	13.46
2	1	2 (20)	2 (400)	15.24
3	1	3 (30)	3 (500)	15.71
4	2 (2:0.6)	1	2	27.6
5	2	2	3	36.15
6	2	3	1	36.82
7	3 (2:0.9)	1	3	34.52
8	3	2	1	52.22
9	3	3	2	33.48
K1	44.41	75.68	102.50	
K2	100.66	103.62	76.41	
K3	120.23	86.00	86.38	
k1	14.80	25.23	34.17	
k2	33.55	34.54	25.47	
k3	40.08	28.67	28.79	
R	25.27	9.31	8.70	
Factors	A_3_	B_2_	C_1_	
Optimal condition	A_3_B_2_C_1_			
